# Investigation of Potential Targets for Bupleuri Radix in the Treatment of Subacute Thyroiditis

**DOI:** 10.1155/ije/7460422

**Published:** 2025-03-11

**Authors:** Xiaoli Lei, Lina Lv, Yongsheng Ma, Chenda Zhang, Li Li, Yanqin Huang

**Affiliations:** ^1^The First Clinical Medical College, Shandong University of Traditional Chinese Medicine, Jinan, China; ^2^Chronic Disease Center, Taian Second Hospital of Traditional Chinese Medicine, Taian, China; ^3^Department of Endocrinology, Affiliated Hospital of Shandong University of Traditional Chinese Medicine, Jinan, China

**Keywords:** Mendelian randomization, network pharmacology, Radix Bupleuri, subacute thyroiditis

## Abstract

**Objective:** This study aimed to reveal the underlying mechanism of Radix Bupleuri in the treatment of subacute thyroiditis (SAT).

**Methods:** Radix Bupleuri, a key herb in traditional Chinese medicine (TCM) for over two millennia, is widely used to treat exterior syndromes, dissipate heat, harmonize liver Qi, and invigorate Yang–Qi. It is also commonly prescribed for SAT. To understand its molecular mechanisms, we collected active components and their targets from databases such as TCMSP, SwissADME, and Swiss TargetPrediction. Radix Bupleuri SAT genes were sourced from GeneCards, OMIM, and DisGeNET databases. By analyzing these data sources together, we identified the relevant ingredients of Radix Bupleuri for treating SAT. Using Cytoscape 3.7.1 software, we selected core proteins to construct an ingredient–target–pathway network. Molecular docking and visualization were performed with AutoDock Vina and Discovery Studio tools, respectively. The stability of the binding model was confirmed through molecular dynamics (MD) simulation techniques. To further validate the causal relationship between CDK4 gene in the GWAS database and SAT in the FinnGen database, we conducted a two-sample Mendelian randomization analysis.

**Results:** Through active ingredient–target network screening, 8 key active ingredients and 10 core targets were identified. The strongest binding was observed between cubebin/quercetin and CDK4 with a binding energy of ≤ −9.2 kcal·mol^−1^, whereas isorhamnetin/quercetin showed stable binding with PIK3CA. The stability of cubebin binding with CDK4 was confirmed through MD simulation, indicating that CDK4 potentially modulates SAT by regulating cell cycle progression, cellular proliferation, immune responses, and inflammatory reactions.

**Conclusion:** Radix Bupleuri may affect SAT by its action on CDK4, providing scientific basis for further clinical application of Radix Bupleuri in the treatment of SAT.

## 1. Introduction

Subacute thyroiditis (SAT), also known as granulomatous thyroiditis or De Quervain's thyroiditis [1], has an annual incidence of 9100 cases per 40,000 individuals. It is a self-limiting inflammatory thyroid disease that may last for 2–7 months [2]. It is possibly of viral origin and is typically associated with thyroid pain and various systemic symptoms [3]. The typical clinical manifestations include thyroid enlargement and pain in the thyroid area, with possible radiation of pain, along with fever, sweating, palpitations, and other systemic symptoms. SAT usually progresses through several stages: hyperthyroidism, which is the typical presentation, followed by a euthyroid state, then hypothyroidism, and finally restoration of normal thyroid function. Each stage lasts for several weeks [4]. If left untreated, it can lead to significant discomfort or even complete exhaustion for weeks or months [5]. For the SAT, early management primarily involved nonsteroidal anti-inflammatory drugs (NSAIDs). In more severe cases, glucocorticoid therapy was more frequently applied. However, both NSAIDs and glucocorticoids have limitations in terms of their efficacy as monotherapies. They are associated with side effects, and there is a tendency for relapse after drug discontinuation. Additionally, these treatments can have a significant impact on thyroid function during recovery.

Radix Bupleuri, primarily derived from the roots and rhizomes of *Bupleurum chinense* DC or *B. scorzonerifolium* Willd, is a pivotal ingredient in traditional Chinese medicine (TCM) [6]. The medicinal parts are typically processed into slices and are characterized by their slightly cold nature and bitter taste, associating them with the liver and gallbladder meridians in TCM [7]. Radix Bupleuri is celebrated for its capabilities to reduce fever, disperse liver Qi stagnation, and uplift Yang–Qi.

Modern pharmacological research has revealed a broad spectrum of beneficial activities associated with Radix Bupleuri. Its complex chemical composition endows it with a wide array of properties, including antioxidant, antiviral, cardioprotective, hepatoprotective, nephroprotective, analgesic, antifibrotic, anticancer, and antidepressant effects [8]. These diverse effects are attributed to its various secondary metabolites. To date, over 100 glycosylated saikosaponins have been isolated and identified from Radix Bupleuri, many of which exhibit biological activity both in vitro and in vivo [9].

Radix Bupleuri has a long-standing reputation for treating thyroid diseases and remains a preferred choice for the clinical management of SAT [10]. It is extensively used to address the underlying pathogenesis of liver depression and Qi stagnation in SAT, effectively dispersing liver Qi stagnation and resolving depression, thereby demonstrating significant clinical efficacy [11]. A statistical analysis conducted by Lu and colleagues using the Traditional Chinese Medicine Inheritance Support System software revealed that Radix Bupleuri is the most frequently used herb in the treatment of SAT based on data from the CNKI database [12]. Formulas with Radix Bupleuri as the primary ingredient have not only shown efficacy in treating various thyroid diseases but are also associated with a lower incidence of adverse events according to a meta-analysis [13]. Therefore, reviewing and summarizing the mechanisms of Radix Bupleuri in the treatment of SAT is of significant importance for further research and development.

The objective of this study was to investigate the potential therapeutic targets of Bupleuri Radix in the treatment of SAT. Leveraging system biology principles, network pharmacology employs network analysis and various methodologies to elucidate the intricate relationships between drug targets and diseases. This approach offers a novel framework for predicting the molecular mechanisms underlying TCM, which encompasses multiple active ingredients, relevant targets, and associated signaling pathways [14]. The simulation of ligand–receptor protein interactions through computational platforms is referred to as molecular docking and molecular dynamics (MD) simulation techniques [15, 16]. Mendelian randomization (MR) serves as an effective approach that uses genetic variation as an instrumental variable to identify causal relationships between exposure and disease [17]. This study constructed an “ingredients–targets–disease” network for Bupleuri Radix using network pharmacology. Enrichment analyses via Gene Ontology (GO) and Kyoto Encyclopedia of Genes and Genomes (KEGG) were conducted to investigate target functions and signaling pathways. Molecular docking and dynamics simulations evaluated binding affinities between key components and significant targets, whereas MR was used to examine the causal relationship between core targets and SAT.

## 2. Methods

### 2.1. Screening of Bupleuri Radix Components

To identify the active ingredients in Radix Bupleuri, we used the Chinese Medicine Information System Pharmacology Data Analysis Network Platform (https://old.tcmsp-e.com/tcmsp.php). The active ingredients were selected based on the Traditional Chinese Medicine Systems Pharmacology (TCMSP) database algorithm, which required an oral bioavailability (OB) score greater than 30% and a drug likeness (DL) score greater than 0.18. Next, the SwissADME tool (https://www.swissadme.ch/) was used to further screen the active ingredients based on the Lipinski, Ghose, Veber, Egan, and Muegge criteria. We selected only those ingredients that met at least three of the criteria. After identifying the main active ingredients of Radix Bupleuri, we collected and saved their detailed information.

### 2.2. Prediction of Bupleuri Radix's Related Targets

Active ingredients of Radix Bupleuri were retrieved from PubChem (https://pubchem.ncbi.nlm.nih.gov), and their corresponding SMILE numbers were obtained. The Swiss TargetPrediction database (https://swisstargetprediction.ch/) was used for target prediction based on compound structures. A probability threshold of > 0.1 was set for target selection, and duplicate targets were removed.

### 2.3. Screening of Potential Disease-Related Target for SAT

Using “subacute thyroiditis” as the search term, the GeneCards database (https://www.genecards.org), the OMIM database (https://www.omim.org/), and the DisGeNET database (https://www.disgenet.org/) were searched to identify targets related to SAT. The targets obtained from these three databases were merged, and duplicates were removed.

### 2.4. Construction of the Network of Active Ingredients in Bupleuri Radix–SAT Targets

The intersection of the active ingredient targets of Radix Bupleuri and the targets related to SAT was taken. The R 4.2.2 software was used to draw a Venn diagram. The intersection targets were used to construct a protein–protein interaction (PPI) network model using the String database (https://string-db.org). The biological species was set as “Homo sapiens,” and the nodes without connections were hidden. The minimum interaction score was set as “highest confidence” (> 0.9), and other settings were kept as default. The PPI network was obtained and visualized using Cytoscape 3.7.1 software.

### 2.5. Enrichment Analysis of GO and KEGG

Language packages for GO and KEGG enrichment analysis were downloaded and installed into R 4.2.2 software from the Bioconductor database (https://www.bioconductor.org/). GO and KEGG pathway enrichment bubble plots, bar plots, and corresponding pathway diagrams were drawn using R 4.2.2 software.

### 2.6. Network Diagram of Active Ingredients—Bupleuri Radix–SAT Targets–Pathways

The relationships between the active ingredients of the drug, the intersection targets, and the pathways enriched by the intersection targets were organized. The results were imported into Cytoscape 3.7.1 software to draw a network diagram of active ingredients–targets–pathways and visualize it. The cytoHubba plugin and network topological parameters (node degree, betweenness centrality, and closeness centrality [CC]) in Cytoscape 3.7.1 were used for screening active ingredients for further analysis.

### 2.7. Molecular Docking

In the previous step, we obtained the key active ingredients of the drug and then docked them with the functional targets. We obtained the chemical structure of the active ingredients from PubChem [18] and the protein structure of the functional targets in PDB format from the PDB database (https://www.rcsb.org/). To perform the molecular docking process, we used AutoDock Vina [19] software and then optimized the results using Discovery Studio software. During the process, we used the active ingredients as ligands and the proteins translated from the target genes as receptors. A lower binding energy between the ligand and receptor indicates a more stable binding.

### 2.8. MD Simulations

The resulting proteins were separated from small-molecule ligands. Small-molecule force field files were generated using the antechamber tool in AmberTools and converted to GROMACS force field files with ACPYPE. The Generalized Amber force field was used for small molecules, whereas the Assisted Model Building with Energy Refinement and transferable intermolecular potential with 3 points (TIP3P) water model were applied for proteins. Protein and ligand files were then merged to construct the simulation system of the complex.

MD simulations were performed using the GROMACS 2022 program under constant temperature and periodic boundary conditions. During the MD simulations, all involved hydrogen bonds were constrained using the *linear constraint solver* algorithm with an integration step size of 2 fs. Electrostatic interactions were calculated using the particle-mesh Ewald method, and the cutoff value was set to 1.2 nm. The nonbonded interaction cutoff value was set to 10 A and updated every 10 steps. The simulated temperature was 298 K with the V-rescale temperature coupling method, and the pressure was 1 bar using the Berendsen method. At 298 K, a 100-ps, NVT and NPT equilibrium simulation was conducted, followed by a 100-ns MD simulation on the complex system. The conformation was saved every 10 ps. After the simulations, the simulated trajectories were analyzed using VisualMD and PyMOL and mechanics Poisson–Boltzmann surface area (MMPBSA) between the protein and small-molecule ligands using the program g_mmpbsa for binding free energy analysis.

### 2.9. Two-Sample Randomized Mendelian Analysis

The small molecules cyclin-dependent kinase 4 (CDK4) and SAT were selected for a two-sample MR analysis. Data on CDK4 were obtained from the GWAS catalog database (https://www.ebi.ac.uk/gwas/) with an ID of GCSTGCST90002346, comprising 460,935 cases of European descent. The FinnGen database (https://www.finngen.fi/en) provided data on 1050 patients with SAT and 349,717 control cases. Causality analysis was performed using MR Egger, weighted median (WME), inverse variance weighting (IVW), simple mode (SM), weighted mode (WM), and two-sample MR methods. Single-nucleotide polymorphisms (SNPs) served as instrumental variables in the analysis. The selection criteria for each instrumental variable associated with exposure factors (core target genes) were as follows:

1. Correlation hypothesis—SNPs exhibited strong correlation with core target genes;
2. Exclusivity hypothesis—SNPs showed no correlation with outcome factors; and
3. Independence hypothesis—SNPs had no association with confounding factors.

The two-sample package in R software version 4.3.2 was employed to conduct the MR analysis.

## 3. Results

### 3.1. Identification of Active Ingredients and Related Targets of Bupleuri Radix

Nine main active ingredients have been identified in Radix Bupleuri, including isorhamnetin, kaempferol, cubebin, octalupine, sainfuran, (+)-anomalin, petunidin, and quercetin (refer to Table 1). After removing duplicates, 315 targets were obtained.

### 3.2. Acquisition of Disease Targets

After searching the GeneCards, OMIM, and DisGeNET databases, the disease targets were combined and duplicates were removed, resulting in 1268 unique targets.

### 3.3. Construction of Bupleuri Radix Active Ingredient–SAT Target Network

**Figure 1:**
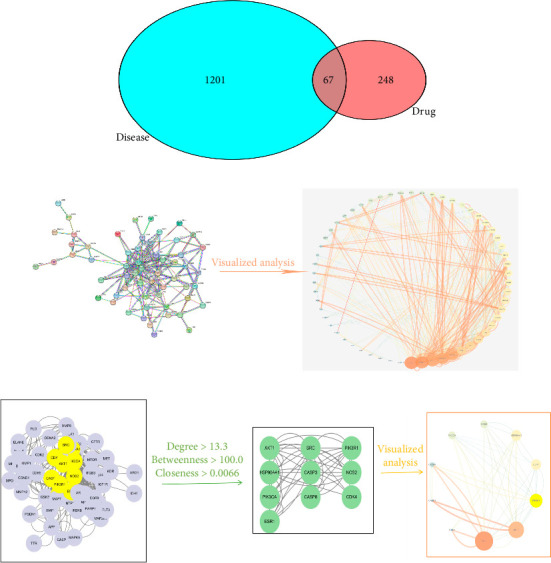
(a) Intersection of Radix Bupleuri–SAT targets. (b) PPI network diagram. (c) Core target topological network.

A total of 67 intersection targets were found when the active ingredients in Radix Bupleuri were compared to SAT targets. These targets were visualized in a Venn diagram (Figure 1(a)) and used to create a PPI network model in the STRING database. The network consisted of 58 nodes and 386 edges, with node size and color reflecting degree value and edge thickness and color reflecting combined score value (Figure 1(b)). Nodes were analyzed for various parameters, with an average between centrality (BC) value of 100.03, average CC value of 0.0067, and average degree centrality (DC) value of 13.31. Nodes with parameter values greater than the average were highlighted. Then, topological analysis was carried out, and 10 core nodes were extracted, namely AKT serine/threonine kinase 1 (AKT1), phosphatidylinositol-4,5-bisphosphate 3-kinase catalytic subunit alpha (PIK3CA), caspase-3 (CASP3), nitric oxide synthase 2, inducible (NOS2), heat shock protein 90 kDa alpha cytosolic, class A member 1 (HSP90AA1), SRC proto-oncogene, non–receptor tyrosine kinase (SRC), estrogen receptor 1 (ESR1), phosphatidylinositol 3-kinase regulatory subunit 1 (PIK3R1), caspase-8 (CASP8), and CDK4, which were taken as the core targets for further analysis (Figure 1(c)).

### 3.4. Enrichment Analysis Was Conducted for the Active Components of Bupleuri Radix in SAT Targets, Focusing on GO Functions and KEGG Pathways

**Figure 2:**
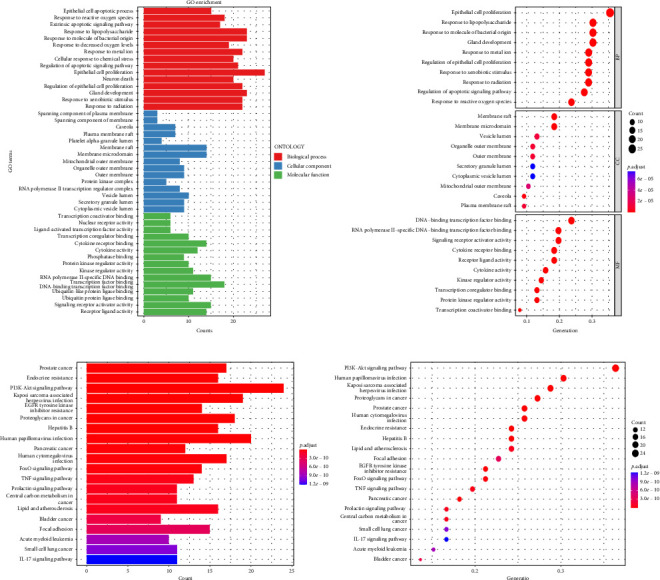
(a) Functional enrichment analysis for intersectional target genes in Radix Bupleuri–SAT. (b) KEGG pathway enrichment analysis of the intersection targets between Radix Bupleuri and SAT.

Using the language packages in the Bioconductor database for GO and KEGG, and with the assistance of the R language, we were able to obtain GO annotations for 1832 biological processes (BPs), 35 cellular components (CCs), and 103 molecular functions (MFs) with a *p* value < 0.05 and adjusted *q* value < 0.05 (Figure 2(a)). Then, we selected the top 10 pieces of information for each category and obtained a bar graph and bubble graph of the main GO functions. Through GO enrichment analysis, we found that the 1832 BPs were mainly related to epithelial cell proliferation, response to lipopolysaccharide, response to molecules of bacterial origin, gland development, and other processes. The 35 CCs were mainly related to the membrane raft and membrane microdomain. Furthermore, the 103 MFs were primarily involved in DNA-binding transcription factor binding, RNA polymerase II–specific DNA-binding transcription factor binding, signaling receptor activator activity, cytokine receptor binding, and other functions (Figure 2(a)).

With the help of R language, the main KEGG functional bar plot and bubble plot could be obtained. Among them, there were 143 KEGG analysis results with *p* value < 0.05 and *q* value < 0.05 after correction. The top 20 results with significant differences in each visualization are shown in Figure 2(b). The signal pathways involved in the cross-targets of Radix Bupleuri and SAT mainly include phosphoinositide 3-kinase—protein kinase B (PI3K-Akt) signaling pathway, human papillomavirus infection, Kaposi's sarcoma-associated herpesvirus infection, human cytomegalovirus infection, prostate cancer, human cytomegalovirus infection, endocrine resistance, hepatitis B, epidermal growth factor receptor (EGFR), tyrosine kinase inhibitor resistance, pancreatic cancer, FoxO signaling pathway, tumor necrosis factor (TNF) signaling pathway, and central carbon metabolism in cancer (Figure 2(b)).

### 3.5. Active “Ingredients–Targets–Signaling” Pathway Network

**Figure 3:**
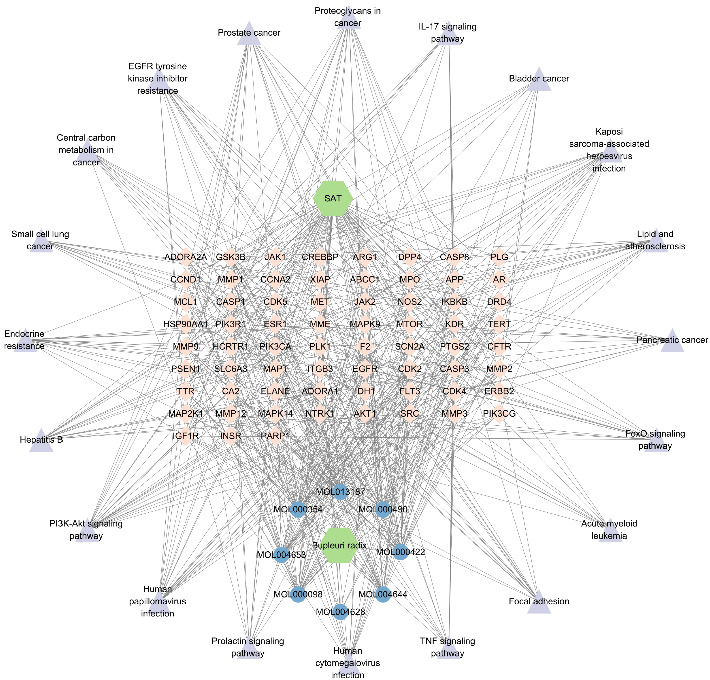
Network of “active ingredients–targets–signal pathways” of Bupleuri radix.

The corresponding relationships between 8 active ingredients, 67 intersecting targets, and the top 20 significantly enriched pathways were organized and imported into Cytoscape 3.7.1 software for visualization. The network of “active ingredients–targets–signaling pathways” for Radix Bupleuri (Figure 3) included eight active ingredients: MOL000354 isorhamnetin, MOL000422 kaempferol, MOL013187 cubebin, MOL004628 octalupine, MOL004644 sainfuran, MOL004653 (+)-anomalin, MOL000490 petunidin, and MOL000098 quercetin. These were represented as core active ingredients (blue circular targets at the bottom). There were also 67 intersecting targets (pink diamond-shaped targets in the middle) and the top 20 enriched pathways (purple triangular targets on the periphery) (Figure 3).

### 3.6. Molecular Docking

**Figure 4:**
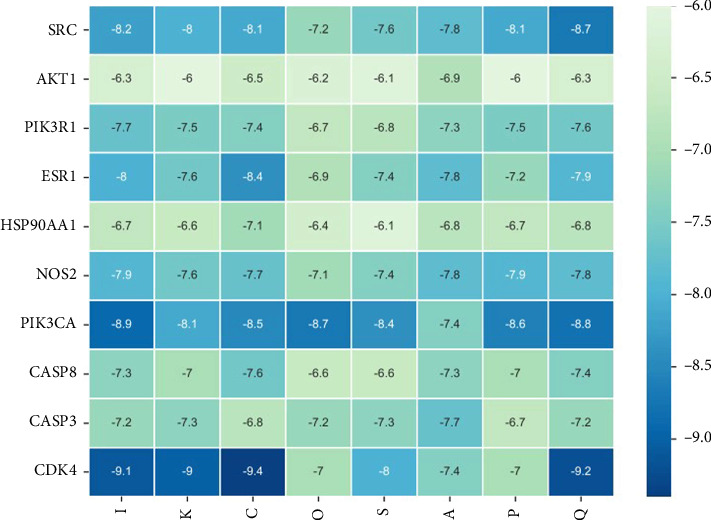
Heat map of target molecule docking. *Note:* I, isorhamnetin; K, kaempferol; C, cubebin; O, octalupine; S, sainfuran; A, (+)-anomalin; P, petunidin; Q, quercetin.

**Figure 5:**
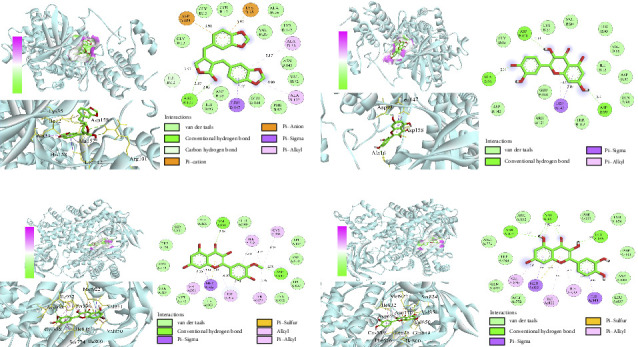
(a) Cubebin and CDK4. (b) Quercetin and CDK4. (c) Isorhamnetin and PIK3CA. (d) Quercetin and PIK3CA.

Using AutoDock Vina, the eight active ingredients were docked with 10 core proteins, as shown in Table 2. Based on Table 2, a molecular docking heat map of core targets was generated in the bioinformatics platform (Figure 4). It is widely accepted that a binding energy of less than −5 kcal·mol^−1^, as determined by molecular docking, suggests a significant binding affinity between the target and the compound; furthermore, lower binding energies correlate with increased stability of their interaction [20]. AutoDock Vina and Discovery Studio software were used for visualizing the docking results. The lower the binding energy, the more stable the compound. Based on this, the most stable binding was observed between CDK and cubebin, as well as quercetin (see Figures 5(a) and 5(b)). The second most stable binding was observed between PIK3CA and isorhamnetin, as well as quercetin (see Figures 5(c) and 5(d)).

### 3.7. MD Simulations

Next, we choose to combine the most stable cubebin and CDK4 to continue the MD simulations.

#### 3.7.1. Stability Analysis of the Complex

**Figure 6:**
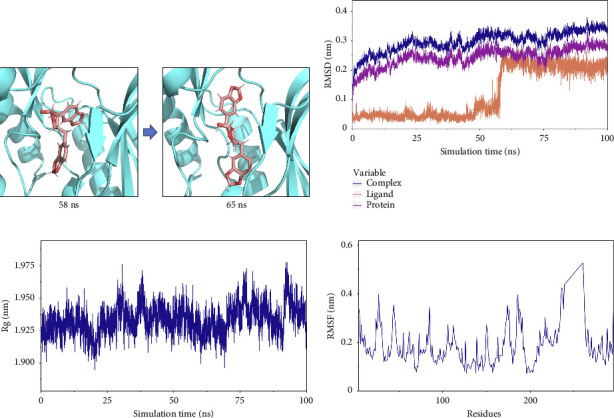
(a) Conformational change in small molecule ligand. (b) RMSD of complex, protein, and small-molecule ligand. (c) The Rg of the complex. (d) RMSF of proteins in the complex.

The root-mean-square deviation (RMSD) quantifies the average magnitude of atomic deviations between a given conformation and the target conformation at a specific time point, serving as a critical metric for assessing system stability.  Figure 6(a) illustrates that the small molecule experienced a conformational change between 58 and 65 ns, leading to a notable increase in RMSD during this interval. Meanwhile, the RMSD values for both the complex and the protein gradually stabilized, indicating that the complex progressively attained a stable state (referred to as Figure 6(b) in the manuscript). The radius of gyration (Rg) is an effective parameter for describing changes in the overall structure and characterizing the compactness of the protein structure. A greater change in Rg signifies a more expanded system. As shown in the figure, the overall Rg of the complex remained stable, suggesting that the overall structure of the complex maintained its stability (Figure 6(c)). The root-mean-square fluctuation (RMSF) measures the flexibility of amino acid residues within a protein (Figure 6(d)).

In this subsection, through the analysis of the data of RMSD and Rg, we determined the stability of the complex during the simulation, and to clarify the exact mechanism, further analysis of the binding between small molecules and proteins is needed.

#### 3.7.2. Analysis of Small-Molecule-Binding Protein Stability

**Figure 7:**
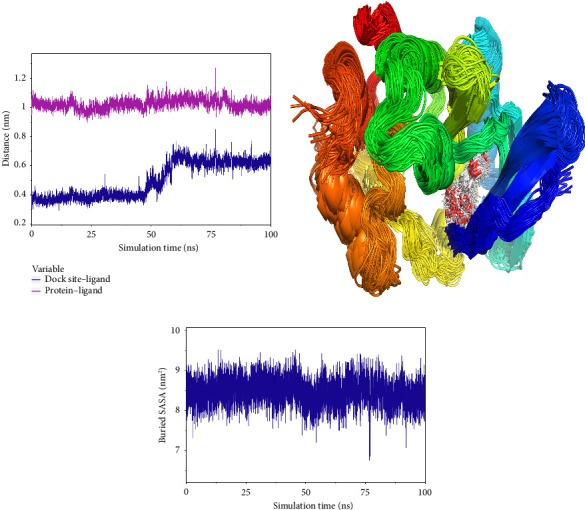
(a) Spacing of binding sites for small molecules on the protein surface (dock site–ligand). (b) Simulated conformation superposition, and in the figure, the winning black circle is the position of the small-molecule superposition conformation. (c) The embedded area between small molecules and proteins (buried SASA).

To analyze the state of small molecules on the protein surface, we first identified their initial docking sites and evaluated the distances between the centers of the residues and the small molecules. Additionally, we analyzed the distance between the small molecules and the protein center. By examining these distances, we were able to infer the binding state of the small molecules with the protein.  Figure 7(a) shows that the position of the small molecule relative to the protein center remained stable, indicating that the small molecule did not dissociate from the protein. The distance between the small molecule and its initial binding site changed during the 50–65-ns interval, which was attributed to conformational adjustments in the small molecule that altered its center of mass, thereby leading to changes in the distance from the initial binding site (Figure 7(a)). By combining the small molecules with the simulated protein conformations, the resulting complex conformations remained near the initial binding site, demonstrating high stacking stability. This indicates consistent and stable binding of the small molecules to the protein throughout the simulation (Figure 7(b)). The buried solvent-accessible surface area (SASA) of small molecules embedded in proteins reflects the size of the binding interface. Analysis of the buried SASA showed that it remained stable during the simulation, further confirming the stable binding of small molecules to the protein (Figure 7(c)).

In this subsection, the stable binding of small molecules to the complex was demonstrated by analyzing the centroid distance between small molecules and proteins, analysis of complex trajectory stacking, and embedding area.

#### 3.7.3. Analysis of Hydrogen Bond Interactions Between Small Molecules and Proteins

**Figure 8:**
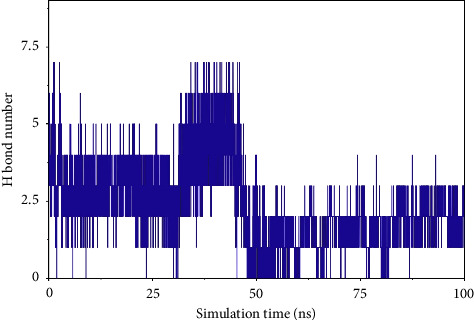
Number of hydrogen bonds (H bond number).

Hydrogen bonding is an important force for protein and ligand binding. Hydrogen bonds are related to the electrostatic interactions and can reflect the strength of the electrostatic interaction. According to the figure, the number of hydrogen bonds between small molecules and proteins was small, and they were mainly distributed between 1 and 3 molecules after 65 ns (referred to as Figure 8).

#### 3.7.4. Analysis of the Interaction of Small Molecules and Protein Binding

**Figure 9:**
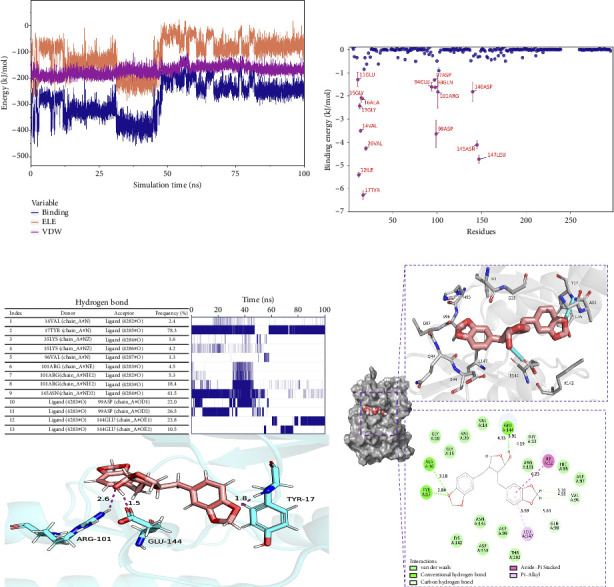
(a) VDW and ELE between small molecules and proteins. (b) Amino acid binding energy contribution. (c) Analysis of hydrogen bond stability. (d) Interaction between the protein and the small molecules.

The van der Waals (VDW) force and the electrostatic interaction force between the complex small molecule and the protein were calculated to analyze the binding force changes during the simulation. Where VDW is the VDW force and hydrophobic interactions, ELE is the electrostatic interaction, and binding is the sum of VDW and ELE, which can represent the binding energy of small molecules with the protein without considering the solvation effect. According to the figure, before 50 ns, the VDW in the complex was caused by the adjustment of small molecules. After 65 ns, the stability of VDW and ELE, especially ELE, became more stable between 80 and 100 ns, indicating a stable binding force between small molecules and proteins, and small molecules could bind stably (Figure 9(a)).

Considering solvation energy, we selected stable complex trajectories based on RMSD, Rg, distance, buried SASA, and interaction energy. Using the MMPBSA method, we calculated the binding energy terms as shown in the table. In the table, ΔEele is the electrostatic interactions between the small molecule and the protein; ΔEvdw is the VDW interactions; ΔEpol is the polar solvation energy (electrostatic potential); ΔEnonpol is the nonpolar solvation energy (hydrophobic interactions); ΔEMMPBSA is the sum of ΔEele, ΔEvdw, ΔEpol, and ΔEnonpol; and ΔGbind is the sum of ΔEMMPBSA and -TΔS.

Analysis of Table 2 reveals that VDW interactions (ΔEvdw) are significantly higher than electrostatic interactions (ΔEele) and hydrophobic interactions (ΔEnonpol). In particular, ΔEvdw is 4.3 times greater than ΔEele and 8 times greater than ΔEnonpol. VDW forces play a predominant role, while electrostatic interactions contribute secondarily, and hydrophobic interactions provide supplementary effects. These findings indicate a high binding energy and strong affinity between the small molecule and the protein.

The binding energy ΔEMMPBSA was decomposed to evaluate the contribution of each amino acid residue. Key residues with significant contributions include TYR17, ILE12, LEU147, ASN145, VAL20, and ASP99, as shown in Figure 9(b). The predominance of hydrophobic amino acids over charged ones suggests that electrostatic interactions do not play a dominant role in the binding energy. These results underscore the critical role of these amino acids in mediating small molecule–protein interactions.

To analyze the hydrogen bond interactions and their stability between proteins and small molecules, we examined the frequency of these interactions. As illustrated in Figure 9(c), only hydrogen bond pairs with a frequency exceeding 1% were selected for further analysis. The left panel presents the acceptor, donor, and frequency of each hydrogen bond pair, whereas the right panel displays the duration of existence for each corresponding pair. Vertical lines indicate the presence of hydrogen bonds; denser lines suggest greater stability. From this figure, it is evident that relatively stable hydrogen bond pairs exist between proteins and small molecules; for instance, the hydrogen bond frequency between TYR17 and its ligand reaches an impressive 78.3%, indicating a highly stable interaction between the small molecule and TYR17.

We selected the final frame of the simulation to analyze its structure and interactions, as shown in Figure 9(d).

Conclusion: (1) Stability: During the simulation, the small molecules bind to the initial binding site, and the small molecules stabilize the binding to the protein; (2) hydrogen bonds: Small molecules form a small number of hydrogen bonds to the protein; (3) binding energy: Gibbs binding free energy of both 76.294 ± 0.847 kJ/mol, high binding energy, and high affinity of small molecules to the protein; (4) binding energy composition: Complex VDW force interaction is higher than that of electrostatic interaction and hydrophobic interaction; VDW force plays a major role, electrostatic interactions play a secondary role, and hydrophobic interactions play a complementary role.

### 3.8. Results of Two-Sample MR Analysis

**Figure 10:**
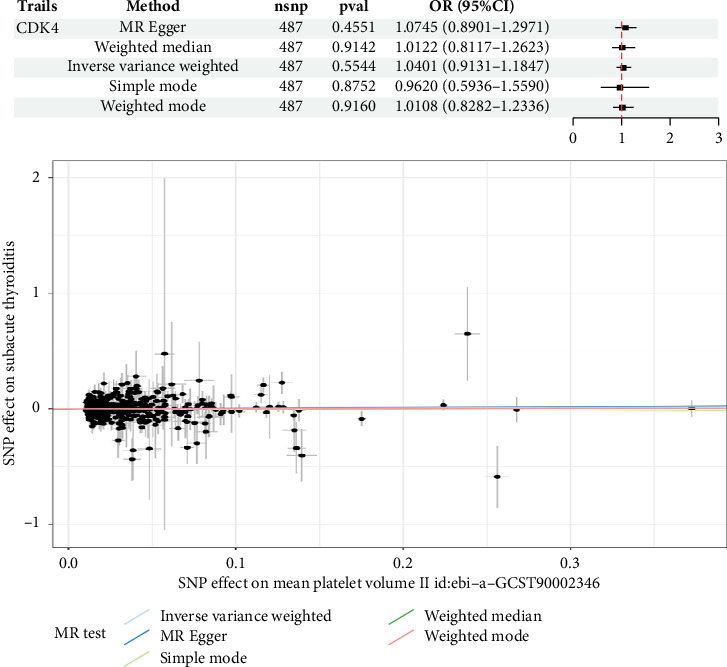
MR analysis results.

Results of the two-sample MR analysis revealed that small-molecule CDK4 was used as the exposure factor and SAT as the outcome variable, with *p* < 5.0 × 10^−8^. After filtering conditions were applied, 503 SNPs were included as instrumental variables after excluding linkage disequilibrium (*r*^2^ = 0.001, kb = 10,000); 16 SNPs with palindromic or incompatible alleles were deleted; and finally, 487 SNPs were identified for MR analysis, among which *F* values were all > 10. IVW was selected as the main analysis method [21]. Although there was a correlation trend between CDK4 and subthyroiditis, this correlation did not achieve statistical significance (OR: 1.0401, 95% CI: 0.9131–1.1847, *p*=0.5544) (referred to as Figure 10).

## 4. Discussion

Radix Bupleuri exhibits multiple functions, including antipyretic, anti-inflammatory, immunomodulatory effects, inhibition of tumor growth, protection of liver cells, and alleviation of depressive symptoms [22, 23]. According to statistical data, Radix Bupleuri is the most frequently used herb in prescriptions for the treatment of SAT in China [24]. Research indicates that the efficacy of Radix Bupleuri combined with Western medicine for treating SAT significantly surpasses that of Western medicine alone. This improvement is primarily reflected in enhanced total effective rates, reduced recurrence rates, and effective alleviation of symptoms such as thyroid pain, hyperhidrosis, hand tremors, fatigue, palpitations, sore throat among patients, and a decrease in erythrocyte sedimentation rate [25, 26]. Nanlian et al. [13] conducted a meta-analysis comparing the effects of Radix Bupleuri prescriptions with hormone therapy for SAT. Their findings revealed that compared to hormone therapy, Radix Bupleuri prescriptions demonstrate significant advantages in improving total effective rates while shortening treatment duration and minimizing adverse reactions [11].

Kaempferol has been shown to have many effects, such as anticancer, antibacterial, antifungal, and antiprotist activities [27]. Isorhamnetin also has anti-inflammatory, antioxidant, organ-protective, and antiobesity effects, which are achieved through the regulation of PI3K/AKT/PKB, nuclear factor kappa-light-chain enhancer of activated B cells (NF-κB), mitogen-activated protein kinase (MAPK) signaling pathways, and the expression of related cytokines and kinases [28]. Cubebin possesses numerous biological actions such as anti-inflammatory, histamine antagonist, antifungal, antileukemic, trypanocidal, antimycobacterial, analgesic, and antispasmodic [29]. (+)-Anomalin element is a potential bioactive molecule in the treatment of common diseases, such as anti-inflammatory and neurodegenerative diseases [30]. The petunidin element has antioxidant, anti-inflammatory, and anticancer effects. It can reduce oxidative stress and alleviate myocardial ischemia–reperfusion injury by inhibiting the production of reactive oxygen species (ROS) by targeting NOX4 [31]. Quercetin is an important flavonoid with various characteristics, such as lowering blood pressure, antihyperlipidemia, antihyperglycemia, antioxidant, antiviral, anticancer, anti-inflammatory, antimicrobial, neuroprotective, and cardioprotective effects [32].

After conducting a thorough analysis using GO and KEGG, it was discovered that Radix Bupleuri's active ingredients can interact with receptors located in various parts of cells. This study further found through GO and KEGG analysis that the active ingredients of Radix Bupleuri may interact with receptors distributed in different parts of the cell, thereby participating in various BPs and regulating multiple pathways. Among them, KEGG suggests that the PI3K-Akt signaling pathway may be a key pathway for Radix Bupleuri to treat SAT. The PI3K/AKT signaling pathway promotes antiapoptosis, anti-inflammation, proliferation, migration, and wound-healing functions in cells [33]. Xiao et al. [34] found that the activation of the PI3K/Akt signaling pathway can delay inflammation, prevent glial scar formation, and promote neural function recovery. Wang, Zhang, and Gong [35] found that the main functions of this pathway include inducing stem cell differentiation and metastasis, promoting cell proliferation, inhibiting cell apoptosis, and regulating tissue inflammation, tumor growth, and invasion.

Through PPI, we found that SRC, AKT1, PIK3R1, ESR1, HSP90AA1, NOS2, PIK3CA, CASP8, CASP3, and CDK4 may be key proteins for treating SAT with Radix Bupleuri. SRC protein kinases are proto-oncogenes that play key roles in cell morphology, motility, proliferation, and survival [36]. AKT1 belongs to serine/threonine kinases and activates the PI3K/ALK signaling pathway, playing a key role in immune cell differentiation, proliferation, and migration, and is involved in the formation of systemic and local inflammation [37]. Activation of the Akt pathway aggregates inflammatory and metabolic signals, regulates macrophage responses, and regulates their activated phenotypes [38]. PI3K regulates many facets of metabolic homeostasis. As a regulatory subunit of PI3K, PIK3R1 plays an important role in the modulation of PI3K activity [39]. The biological effects of PIK3R1 include increased apoptosis, decreased cell division, and reduced insulin-stimulated glucose uptake [40]. ESR1 is a steroid receptor, and there have been reports of SAT presenting as Cushing's syndrome [41]. Whether there is a correlation between the two still needs further research. HSP90AA1 is widely present in various biological cells. When stress occurs, HSP90AA1 can be highly expressed to have anti-inflammatory, antiapoptotic, and antioxidant effects. HSP90AA1 can regulate TNF-α and IL-6 levels, and studies have suggested that inhibiting HSP90AA1 expression can reduce inflammasome activity in mice and reduce the secretion of inflammatory factors [42]. NOS2 actively participates in the inflammatory process and facilitates the synthesis of proinflammatory mediators, such as interleukin-6 (IL-6) and interleukin-8 (IL-8). The molecular synergy between NOS2 and prostaglandin–endoperoxide synthase 2 (PTGS2), also known as cyclooxygenase-2 (COX-2), may represent a primary mechanism underlying the elicited inflammatory response [43]. PIK3CA mediates different processes in cells, such as promoting cell transformation, tumor occurrence and proliferation, and resistance to cell apoptosis [44]. CASP8 is the initiator caspase of extrinsic apoptosis and inhibits necroptosis mediated by RIPK3 [45]. CASP8 represents the molecular switch that controls apoptosis, necroptosis, and pyroptosis and prevents tissue damage during embryonic development and adulthood [46]. CASP3 is involved in the process of cell apoptosis and is also an effector of oxidative stress [47].

CDK4 is a pivotal regulatory protein that plays a crucial role in the cell cycle, particularly during the transition of cells from the G1 phase to the S phase [48]. The activity of CDK4 not only impacts the progression of the cell cycle but also influences cellular dimensions, protein synthesis rate, and subcellular structure formation such as nucleolus, mitochondria, and endoplasmic reticulum [49]. In addition to its involvement in cell cycle regulation, CDK4 also exerts significant influence on immune system function. For instance, it governs the proliferation of regulatory T cells (Tregs) through modulation of the classical retinoblastoma (Rb)-E2F pathway. Suppression of CDK4 may impede Treg proliferation, thereby disrupting immune homeostasis and cellular clearance [50]. Furthermore, deficiency in CDK4 can lead to heightened levels of endogenous DNA damage, which activates the cyclic GMP-AMP synthase (cGAS) stimulator of interferon genes (STING) signaling pathway, subsequently triggering the Type I interferon response; this cascade potentially aids in activating CD8 T cells, thus contributing to immunity [51]. Regarding inflammation, the role played by CDK4 appears multifaceted as although its primary function revolves around cell cycle control, aberrations in cell cycle dynamics and dysregulated cellular proliferation are key factors implicated in inflammation and autoimmune diseases. Consequently, modulating CDK4 activity could exert an impact on inflammatory processes [52] with specific mechanisms varying depending on cell type and tissue environment.

Currently, the etiology and mechanisms underlying SAT remain inadequately understood. The T-cell-mediated immune response to thyroid antigens is believed to play a role in the development of SAT [53]. Existing literature generally indicates that its pathogenesis encompasses genetic predisposition linked to human leukocyte antigens, factors associated with viral infections, and autoimmune disorders [54]. Viral infections can lead to various forms of immune dysfunction, including hyperactive immune responses and immunosuppression, while also causing direct damage to thyroid cells [55]. Moreover, during the COVID-19 pandemic, it has been demonstrated that the SARS-CoV-2 virus can provoke an exaggerated immune response and significantly increase IL-6 levels, potentially worsening the onset of thyroiditis [1].

In thyroid disease, abnormal CDK4 expression may be linked to excessive proliferation and inflammation of thyroid cells [56]. We hypothesize that CDK4 influences SAT through its role in cell cycle control, proliferation, immune regulation, and inflammation. In examining the role of CDK4, the study highlighted that while MR analysis did not establish a genetic causal relationship between CDK4 and SAT, this does not imply that CDK4 is inconsequential in the pathogenesis of SAT. The expression of CDK4 may be modulated by the inflammatory environment present in SAT; for instance, cytokines released by inflammatory cells could directly or indirectly influence CDK4 activity, thereby affecting disease progression at a nongenetic level. Furthermore, the inflammatory milieu in SAT may induce epigenetic modifications that impact CDK4 expression without necessitating genetic variation within the gene itself [57, 58]. Additionally, CDK4 might regulate thyroid cell proliferation and survival through interactions with other proteins involved in the inflammatory response, such as kinases, transcription factors, or cell cycle regulators [59, 60].

Cubebin inhibits cyclooxygenase activity and reduces prostaglandin production [61]. Its mechanism of action closely resembles that observed in the majority of NSAIDs [62], while also downregulating iNOS expression. Furthermore, it exhibits antioxidant properties [63] and demonstrates antiproliferative activity [64]. Cubebin has the potential to mitigate genomic damage [65]. Additionally, cubebin displays anti-inflammatory properties that may alleviate thyroid cell damage induced by subthyroiditis, including the prevention of genomic damage. Moreover, by modulating CDK4 activity, cubebin may facilitate the maintenance of a normal cell cycle, thereby inhibiting the proliferation of damaged cells. In the context of subthyroiditis, cubebin's antioxidant and anti-inflammatory characteristics may protect thyroid cells from oxidative stress and inflammation-mediated DNA damage.

In the future, small-molecule inhibitors targeting CDK4 may represent a novel strategy for the treatment of SAT. Given that CDK4 plays a critical role in the abnormal proliferation and inflammation of thyroid cells, therapeutic approaches aimed at this target are anticipated to mitigate disease severity and decrease reliance on conventional hormone therapy. Personalized medicine: If subsequent studies can identify which patients are most likely to benefit from CDK4 inhibitor treatment, more tailored therapeutic options could be developed. A comprehensive understanding of the mechanisms underlying Radix Bupleuri and its active component cubebin will facilitate the exploration of TCM's potential applications within modern medical frameworks and promote the integration of Eastern and Western medical practices. Targeting specific molecular pathways has the potential to reduce systemic side effects while enhancing patient compliance compared with traditional therapies. Furthermore, emerging treatments for SAT exhibit significant market potential, particularly among populations where conventional therapies prove ineffective or poorly tolerated.

In this study, we explored the potential therapeutic targets of Radix Bupleuri for the treatment of SAT and used advanced pharmacological and bioinformatics methodologies to elucidate the specific mechanisms of action associated with its active constituents. Compared to existing research, our work not only enhances the understanding of Radix Bupleuri's pharmacological effects but also provides a systematic analysis, for the first time, of its critical regulatory points related to SAT. Through an examination of published literature, we identified that this study's contribution lies in clarifying Radix Bupleuri's multifaceted roles in modulating the cell cycle and inhibiting inflammatory responses. This establishes a robust scientific foundation for its clinical application while simultaneously providing a theoretical framework for developing innovative therapeutic strategies targeting subacute SAT.

## 5. Conclusion

Radix Bupleuri is frequently employed in the management of SAT. The principal active compound in Radix Bupleuri, cubebin, may facilitate the maintenance of a normal cell cycle by modulating CDK4 activity, thereby inhibiting the proliferation of damaged cells and safeguarding thyroid cells from oxidative stress and inflammation-mediated DNA damage. Investigating the role of CDK4 in the pathogenesis of this condition elucidates its intricate mechanisms and provides a theoretical foundation for novel therapeutic strategies. For instance, inhibitors targeting CDK4 could represent promising new therapies to curtail the abnormal proliferation of thyroid cells and mitigate inflammation.

## Figures and Tables

**Table 1:** Active ingredients of Bupleuri radix.

Mol ID	Molecule name	OB (%)	DL
MOL000354	Isorhamnetin	49.60	0.31
MOL000422	Kaempferol	41.88	0.24
MOL013187	Cubebin	57.13	0.64
MOL004628	Octalupine	47.82	0.28
MOL004644	Sainfuran	79.91	0.23
MOL004653	(+)-Anomalin	46.06	0.66
MOL000490	Petunidin	30.05	0.31
MOL000098	Quercetin	46.43	0.28

**Table 2:** Binding energy and its composition at steady state (kJ/mol).

Complex	ΔE_vdw_	ΔE_ele_	ΔE_pol_	ΔE_nonpol_	ΔE_MMPBSA_	−TΔS	ΔG_bind_^∗^
Protein–ligand	−181.677 ± 1.067	−41.694 ± 0.194	151.299 ± 0.836	−22.651 ± 0.038	−94.723 ± 0.971	18.429 ± 0.209	−76.294 ± 0.847

⁣^∗^ΔG_bind_ = ΔE_vdw_ + ΔE_ele_ + ΔE_pol_ + ΔE_nonpol_ − TΔS.

## Data Availability

The data presented in this study are openly accessible. The Radix Bupleuri data supporting the findings of this study can be accessed from the TCMSP database (https://old.tcmsp-e.com/tcmsp.php). The SAT data can be found in the GeneCards database (https://www.genecards.org), the OMIM database (https://www.omim.org/), and the DisGeNET database (https://www.disgenet.org/). Data on CDK4 were obtained from the GWAS catalog database (https://www.ebi.ac.uk/gwas/) with an ID of GCSTGCST90002346. MR analysis for relevant SAT data is available in the FinnGen database (https://www.finngen.fi/en).
